# Comparative Study of Lingual Papillae, Lingual Glands and Lyssa of the Tongue of Selected Wild Felids (Carnivora, Felidae) in Biological Aspects

**DOI:** 10.3390/biology12040516

**Published:** 2023-03-29

**Authors:** Karolina Goździewska-Harłajczuk, Karolina Barszcz, Joanna Klećkowska-Nawrot, Pavla Hamouzová, Petr Čížek, Piotr Kuropka, Pavel Kvapil

**Affiliations:** 1 Division of Animal Anatomy, Department of Biostructure and Animal Physiology, Faculty of Veterinary Medicine, Wroclaw University of Environmental and Life Sciences, Kozuchowska 1, 51-631 Wroclaw, Poland; 2 Department of Morphological Sciences, Institute of Veterinary Medicine, Warsaw University of Life Sciences, Nowoursynowska 159, 02-787 Warsaw, Poland; karolina.barszcz@sggw.edu.pl; 3 Department of Physiology, Faculty of Veterinary Medicine, University of Veterinary Sciences Brno, Palackého tř. 1946/1, 612 42 Brno, Czech Republic; hamouzovap@vfu.cz; 4 Department of Anatomy, Histology and Embryology, Faculty of Veterinary Medicine, University of Veterinary Sciences Brno, Palackého tř. 1946/1, 612 42 Brno, Czech Republic; cizekp@vfu.cz; 5 Division of Histology and Embryology, Department of Biostructure and Animal Physiology, Faculty of Veterinary Medicine, Wrocław University of Environmental and Life Sciences, Norwida 25, 50-635 Wroclaw, Poland; piotr.kuropka@upwr.edu.pl; 6 Ljubljana Zoo, Večna Pot 70, 1000 Ljubljana, Slovenia; pavel.kvapil@gmail.com

**Keywords:** tongue, hypercarnivorous diet, Neofelis nebulosa, Panthera leo bleyenberghi, Lynx lynx, Otocolobus manul

## Abstract

**Simple Summary:**

The aim of this study was detailed analyses of the lingual surface, lingual glands and lyssa of the four wild species of Felidae. Macroscopic analysis and stereoscopy were used for the general description of the studied tongues. Light microscopy and scanning electron microscopy were used for detailed analysis of the lingual papillae and lingual glands structure. The intrinsic and extrinsic muscles of the tongue were not analyzed in the present study. In all four species of wild felids, typical mechanical lingual papillae were present. Fungiform and vallate papillae were also observed. The main difference was in the foliate papillae area. Histochemical analysis of the lingual glands showed the dominance of mucous secretion in all four wild species of Felidae. The obtained results broaden the anatomical knowledge regarding the similarities and differences of the papillae on the surface of the tongue as well as the lingual glands in biological aspects. Additionally, since the lyssa was characterized in the following study, this may be an introduction to the further comparative analysis of this interesting structure.

**Abstract:**

Although much attention has been paid in the literature to the morphology of the tongue in various animal species, including some Felidae, it has not yet been described in detail in the vulnerable *Neofelis nebulosa* and *Panthera leo bleyenberghi* and the last concern *Lynx lynx* and *Otocolobus manul*. Therefore, the present study aimed to characterize the features of the tongue surface, lingual glands and lyssa in the above-mentioned four selected wild species of the Pantherinae and Felinae subfamilies. Macroscopic, histological, histochemical and ultrastructural analyzes were used in the present work. Comparative analyzes of the dorsal tongue surface showed the presence of mechanical lingual papillae on five subtypes of filiform papillae on the apex and body and conical papillae on the root of the tongue. Gustatory papillae in the four analyzed species were fungiform papillae and various numbers of vallate papillae. Foliate papillae were absent in *P. leo bleyenberghi* and *L. lynx*, while delicate smooth folds, which were separated by parallel grooves but without taste buds, were present in *N. nebulosa*. The vallate and foliate papillae were accompanied by lingual glands, which produced a serous secretion, whereas the mixed lingual glands of the lingual root were with a predominance of mucus secretions comparable to four captive Felidae species. In the median plane on the ventral surface of the apex under its epithelium and within the muscle fibers, the lyssa was also observed to a varying degree, with the least developed, and thought about the size of the entire tongue, was in *P. leo bleyenberghi*. The lyssa structure in the four species was dominated by adipose tissue. The obtained results contribute knowledge concerning the functional anatomy of the tongue in four selected Felidae species, especially in terms of comparative anatomy.

## 1. Introduction

The family Felidae, composed of two subfamilies *Pantherinae* and *Felinae*, includes species with a notable variation in size but all with a hypercarnivorous diet [1,2]. Their highly specialized predatory habit, together with the relatively young age of this group (Late Miocene), is the cause of the low anatomical disparity of felids compared to other groups of carnivores [1,3]. The felids are considered morphologically homogeneous among carnivores with their adult cranial morphology and function [1,2,4]. This indicates that increasing specialization within the hypercarnivorous niche may constrain subsequent morphological and ecological flexibility [3].

**Figure 1:**
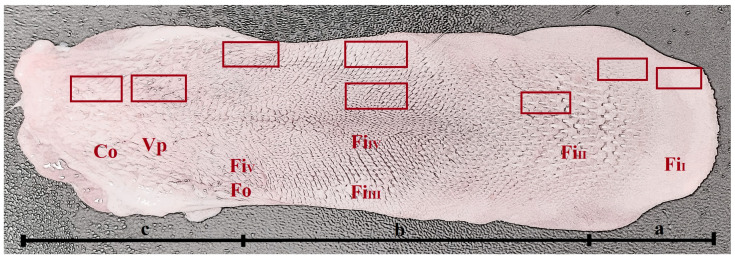
Macroscopic view of the dorsal surface of the tongue of *N. nebulosa.* Division of the tongue into apex (**a**), body (**b**) and root (**c**). See the areas (rectangles) for the collection of samples for histological and histochemical analyses. *Abbreviations*: Co—conical papillae; Fi_I_, Fi_II_, Fi_III_, Fi_IV_ and Fi_V_—types of filiform papillae; Fu—fungiform papillae; Fo—foliate papillae.

The tongue is an important organ of the digestive system and has a significant role in grasping, swallowing and scrolling foods [5]. Meat and tough food consumption in carnivores requires extreme modifications of the skull to withstand the high biomechanical loads imposed by catching and holding live prey and crushing and cracking hard food items, such as bones [6,7]. The tongue must also be adapted to a hypercarnivorous diet as felids use it for the laceration of meat, as well as retaining food within the mouth and the frequent cleaning of fur [8]. Therefore, the tongue of felids is typically densely covered with large mechanical papillae [5,6,9,10,11,12,13,14,15,16,17,18,19,20,21,22,23,24]. These types of papillae especially enable the cat to scrape every piece of meat off a bone and aid in grooming [6]. The appearance of the filiform papillae varies depending on the species and the area of the tongue. In jaguar (*Panthera onca*), the filiform papillae comprise large main papillae and secondary papillae in the apex, large cylinder-like papillae in the anterior part of the body and big conical papillae in the central part of the body [15]. In the Persian leopard (*Panthera pardus saxicolor*), the keratinized filiform papillae are distributed all over the entire dorsal surface of the tongue and contain small processes. They change into a cylindrical shape in the body and a conical shape in the root [18]. The Siberian tiger (*Panthera tigris altaica*) has hemispherical and horny-shaped papillae in the apex, as well as papillae with several pointed processes. The body contains giant club-shaped, conical or bifid and hoe-shaped papillae [13]. On the other hand, the Asian golden cat (*Catopuma temminckii*) has horny-shaped filiform papillae in the lingual apex, large and cylindrical in the anterior part of the body and large and conical papillae in the central part of the body [10]. In the ocelot (*Leopardus pardalis*), the filiform papillae, observed in large numbers in the apex and body, are structurally tapered and have a broader base compared with their apex, and their height increase from one region to another. The conical papillae are in the root [5]. Additionally in the fishing cat (*Prionailurus viverrinus*), the filiform papillae in the lingual apex have several pointed processes, whereas large cylindrical papillae in the anterior part of the body are large and cylindrical, and large and conical papillae are in the central part of the body [14], while in puma (*Puma concolor*), the margins of the apex are surrounded by numerous papillae with a bulky papillary body and a bifurcated tip, and the dorsal surface of the apex contain remarkably pointed papillae with many secondary projections, which emerge from the base of the main papilla. The mechanical papillae of the body contain filiform, longer, cylindrical papillae with blunt tips (in the rostral half of the body) and conical papillae exhibiting a pointed tip (in the caudal part of the body) [6]. In our study, the example of the areas of distribution of mechanical lingual papillae are shown in *N. nebulosa* in Figure 1, but the other examined animals also have a similar distribution of these papillae.

Gustatory papillae with taste buds include fungiform, vallate and foliate papillae, which are observed within the Felidae family species; however, foliate papillae are not developed in the majority of felids [5,6,9,10,11,12,13,14,15,16,17,18,19,20,21,22,23,24]. Adaptations to carnivorous feeding are remarkable even in taste abilities: the taste buds of cats are highly responsive to amino acids and unresponsive to many mono- and disaccharides [25].

Despite the well-known fact that the in some carnivores, the tongue contains a lyssa, whose functional importance is not fully understood [26,27], little attention has been paid to its ultrastructure. Regarding felids, a lyssa composed of adipose tissue with a few striated muscles has been described in the domestic cat [26], whereas a lyssa made of hyaline cartilage surrounded by thin collagen fibers was reported in the Persian leopard [18]. This indicates interspecies differences in the structure of lyssa.

Very little attention has been paid to the lingual salivary gland ultrastructure in felids. A basic description was performed only in the ocelot [5], Persian leopard [18] and lion [19]. Moreover, histochemical analyses of glandular cells with their secretion properties were not performed at all.

This study aimed to describe the macroscopic and microscopic structures of the lingual surface, the features of the lingual glands and the lyssa of the tongue in the clouded leopard (*Neofelis nebulosa*), Katanga lion (*Panthera leo bleyenberghi*), Pallas’s cat (*Otocolobus manul*) and Eurasian lynx (*Lynx lynx*). Another goal of the work was to compare these structures of the tongue with the features of the tongues of other members of the *Pantherinae* and *Felinae* subfamilies and characterize the adaptation of the feline tongue to their hypercarnivorous diet. In addition, the obtained results will contribute to the knowledge of the functional anatomy of the taste organs in vulnerable species (according to the IUCN list) and may be helpful in future comparative studies and research on the adaptation of species to living habitats. The aim of our study was also the explanation of the relationship between the results obtained and the natural environment in which animals are found, their diet and their age.

## 2. Materials and Methods

### 2.1. Collection of the Materials

The research material consisted of 5 tongues derived from 4 different species of undomesticated captive adult felids: Katanga lion (*P. leo bleyenberghi*)—male and female, clouded leopard (*N. nebulosa*), Eurasian lynx (*L. lynx*) and Pallas’s cat (*O. manul*) (Table 1). The material was collected in 2019–2022 at Zoo Wrocław (Poland) and Zoo Ljubljana (Slovenia). Tongues were collected from animals directly post-mortem, and a post-mortem examination revealed no pathological changes in the oral cavity. In addition, the cause of death of the animals was not related to any oral disease. The consent of the Ethics Committee was not required to collect the research material because, according to the law in force in Poland, scientific research covering material obtained post-mortem or from a slaughterhouse is not subject to the consent of the Ethical Committee (The Journal of Laws of the Republic of Poland, the Act of 17 November 2021, on the protection of animals used for scientific or educational purposes). Personal permits were obtained from the district veterinary doctor in Wrocław (Poland) for the collection of the dead material. The numbers of the obtained permits are No. PIW Wroc. UT-45/5/16, No. PIW Wroc. UT- 45/6/16 and No. PIW Wroc. UT-45/8/16.

### 2.2. Macroscopic and Stereoscopic Comparative Analyses

The tongues were rinsed in a saline solution (phosphate buffered saline 0.01 M pH 7.2) (tongue of *L. lynx* was collected directly to the 4% formaldehyde stabilized with phosphate buffer (pH 7.2–7.4)). The storage temperature of samples was between 2 and 8 °C. After the tongues were rinsed and dried, the length, width and thickness of the tongues were measured using digital caliper (with an accuracy of 0.1 mm). The length of whole tongue, the width of apex, body and root as well as the thickness of the apex, body and root were measured using the same method in all studied animals (Figure 1). A Canon EOS 300 camera was used for photographic documentation. Then, the dorsal, ventral and lateral surfaces of tongues were analyzed on their apex, body and root. The location of individual types of lingual papillae was assessed and divided into mechanical and gustatory papillae to compare them with individual species from the Felidae family. Moreover, lyssa was prepared from the medial plane of ventral surface of the apex. The tongue was attached to the pad with pins and was in a completely relaxed state during the analysis. Then, the length of lyssa was measured using the electronic caliper. Consecutive analyzes were performed using a Zeiss Stemi 2000-C microscope (Carl Zeiss, Jena, Germany), and photographic documentation was made with the Axio Vision 4.8 program (Carl Zeiss MicroImaging GmbH, Jena, Germany).

The *m. lingualis proprius* and extrinsic muscles of the tongue were not analyzed in present study.

### 2.3. Light Microscopic Analyses—Histological and Histochemical Studies

Systematic sampling strategies were applied for the collection of the research material (from one half of each tongue). Samples of the different types of lingual papillae from the apex, body and root of the tongue were collected (8 samples from each tongue (Figure 1)), including samples of lingual glands from the root of the tongue based on the same method. The size of each collected sample was 1 cm × 0.5 cm approximately. In addition, slices containing lyssa were taken from each tongue (one sample from each tongue from the middle part of the lyssa).

Tissue sections were placed in 4% formaldehyde stabilized with phosphate buffer (pH 7.2–7.4) for at least 72 h. In the next stage, the collected tissues were dehydrated in a series of alcohol dilutions, and then they were placed in xylene and embedded in paraffin in the form of blocks, which were used for further analysis [28]. The specimens were cut using a Slide 2003 (Pfm AG, Köln, Germany) sliding microtome.

For histological evaluation of the tissues, each specimen was stained using three methods: hematoxylin and eosin (H&E), Azan trichrome and Masson–Goldner trichrome kit. To assess the secretion types of lingual glands, histochemical methods were used: periodic acid-Schiff (PAS), alcian blue pH 1.0 (AB pH 1.0), alcian blue pH 2.5 (AB pH 2.5), periodic acid–Schiff–alcian blue pH 2.5 (PAS-AB pH 2.5) and Hale’s dialyzed iron staining (HDI) [29]. The slides were analyzed using Zeiss Axio Scope A1 light microscope (Carl Zeiss, Jena, Germany). Measurements of individual mechanical and gustatory papillae were performed using Axio Vision 4.8 (Carl Zeiss MicroImaging GmbH, Jena, Germany) software (mean ± SD). Plan-Apochromat 20×/0.45 M27, 40× and EC Plan-Neofluar 63×/0.95 objectives were used for detailed analysis.

### 2.4. Scanning Electron Microscopic Analyses (SEM)

Systematic sampling strategies were applied for the collection of the research material. Tissue samples for SEM [30] were taken from apex, body and root parts of the 5 tongues (8 samples from one half of each tongue). Each sample contained lingual papillae to compare with the studied species of Felidae family.

Samples of lingual papillae were placed in 2.0% glutaraldehyde dissolved in 0.1M phosphate buffer at pH 7.4. Further sample analyses were performed based on methodology of Čížek et al. [31]. SEM samples were dried at the critical point (Bal-tec CPD 030 Critical Point Dryer, Leica Biosystems, Wetzlar, Germany) and then gold-coated (Balzers SCD 040 by current 30 mA for 4 min, Balzers, Lichtenstein). Photographic documentation was obtained using Tescan VEGA TS 5,136 XM (Tescan, s.r.o., Brno-Kohoutovice, Czech Republic) scanning electron microscope in a high vacuum and accelerated voltage 20 kV using an SE detector.

In this publication, all anatomical and histological terms are used based on the terminology of *Nomina Anatomica Veterinaria* (2017) [32] and *Nomina Histologica Veterinaria* (2017) [33].

## 3. Results

### 3.1. Macroscopic and Stereoscopic Comparative Analysis

**Figure 2:**
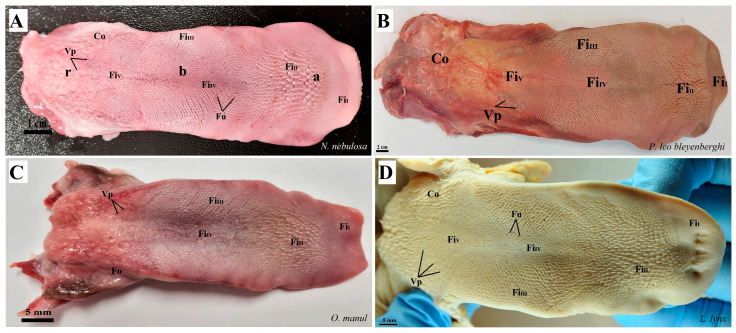
Photograph of the dorsal surface of the tongue of four species of undomesticated Felidae: *N. nebulosa* (**A**), *P. leo bleyenberghi* (**B**), *O. manul* (**C**) and *L. lynx* (**D**). Bar = 2 cm (**B**); bar = 1 cm (**A**); bar = 5 mm (**C**,**D**). Abbreviations: a—apex of the tongue, b—body of the tongue, Fi_I_—filiform papillae subtype I (with caudal orientation of these papillae), Fi_II_—filiform papillae subtype II (with caudal orientation of these papillae), Fi_III_—filiform papillae subtype III (with medio-caudal orientation of these papillae), Fi_IV_—filiform papillae subtype IV (with caudal orientation of these papillae), Fi_V_—filiform papillae subtype V (with caudal orientation of these papillae), Fu—fungiform papilla, r—root of the tongue, Co—small conical papilla, Vp—vallate papilla.

Anatomically, the tongues of the Felidae are divided into the apex, body and root (Figure 2). All tongues in the four species of captive Felidae were elongated, and the lingual apex was mildly rounded. The length, width and thickness of all the tongues are presented in Table 2.

**Figure 3:**
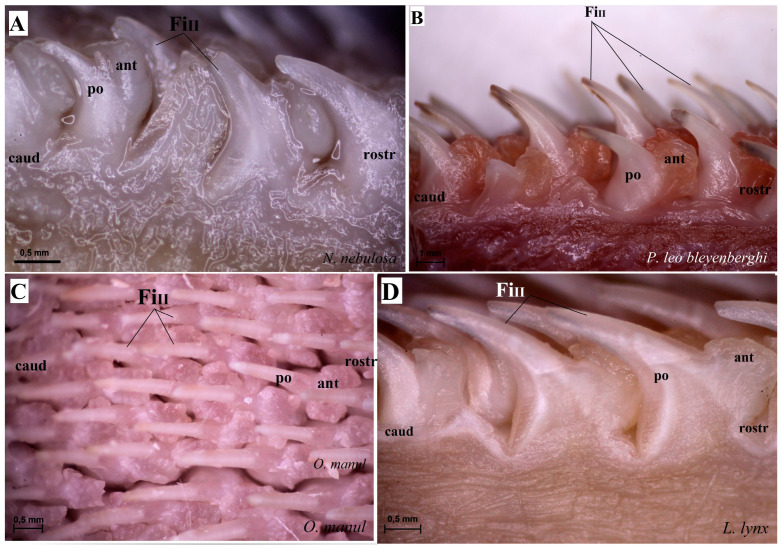
Stereoscopic view of the mechanical lingual papillae from dorsal surface of the rostral part of the tongue of four species of wild Felidae: *N. nebulosa* (**A**), *P. leo bleyenberghi* (**B**), *O. manul* (**C**) and *L. lynx* (**D**). Bar = 0.5 mm (**A**,**C**,**D**); bar = 1 mm (**B**). Abbreviations: ant—anterior part of the mechanical lingual papillae (prominent stratum granulosum), caud—caudal orientation of the mechanical lingual papillae, Fi_II_—filiform papillae subtype II, po—posterior part of mechanical lingual papillae (prominent stratum corneum), rostr—rostral orientation of the mechanical lingual papillae.

**Figure 4:**
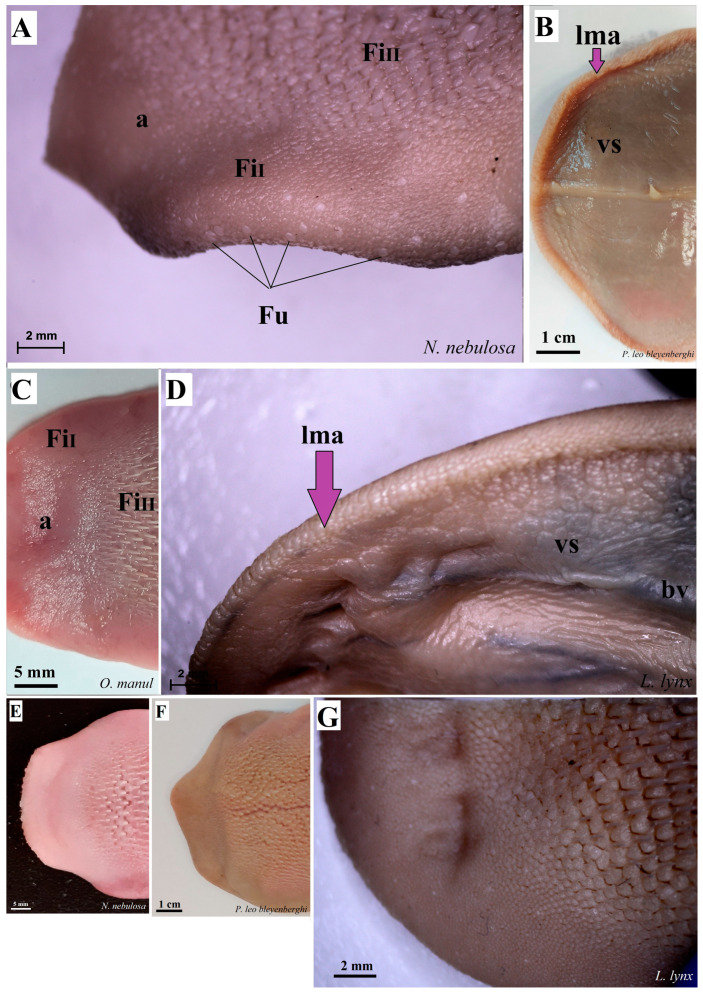
Stereoscopic view of the dorsal surface (**A**,**C**,**E**–**G**) and the ventral surface (**B**,**D**) of the lingual apex of *N. nebulosa* (**A**,**E**), *P. leo bleyenberghi* (**B**,**F**), *O. manul* (**C**) and *L. lynx* (**D**,**G**). Bar = 2 mm (**A**,**D**); bar = 1 cm (**B**); bar = 5 mm (**C**); bar = 2 mm (**A**,**D**). Abbreviations: a—apex of the tongue, bv—blood vessel visible on the ventral surface of the tongue, lma—lateral margin of the lingual apex (purple arrow), vs.—ventral surface of the tongue.

**Figure 5:**
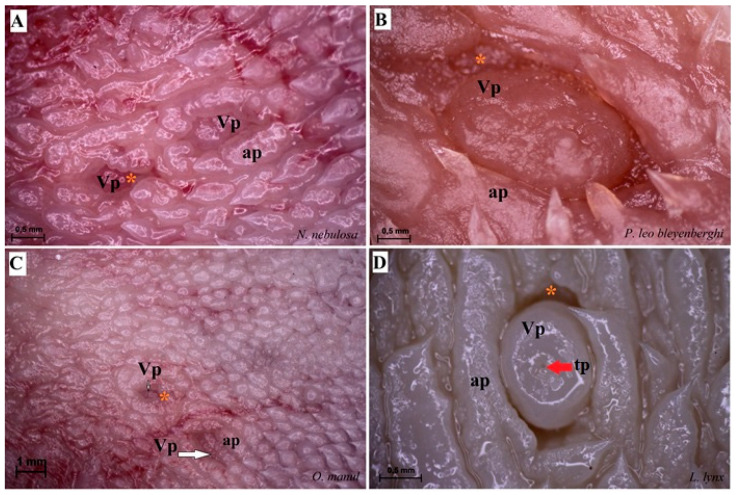
Stereoscopic view of the vallate papillae of the tongue of the four species of undomesticated Felidae: *N. nebulosa* (**A**), *P. leo bleyenberghi* (**B**), *O. manul* (**C**) and *L. lynx* (**D**). (**A**) Two vallate papillae encircled by papillary groove (asterisk). (**B**) Magnification of the elongate vallate papilla with irregular dorsal surface. (**C**) Two vallate papillae among the numerous filiform papillae. (**D**) Magnification of the round shape vallate papilla with well-visible taste pore (red arrow) of the taste bud on the dorsal surface of the vallum of the papilla. Bar = 1 mm (**C**); bar = 0.5 mm (**A**,**B**,**D**). Abbreviations: ap—annular pad with irregular surface, tp—taste pore of the taste bud (red arrow), Vp—vallate papilla (white arrow), * (orange asterisk)—groove of the vallate papilla.

**Figure 6:**
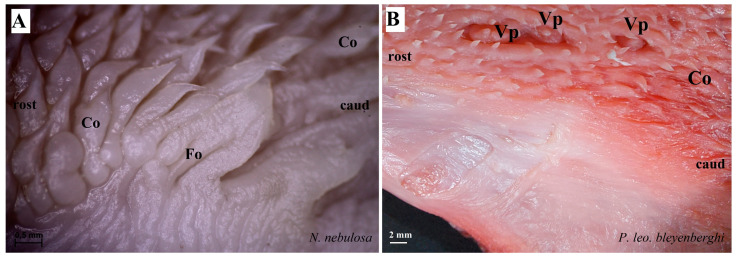

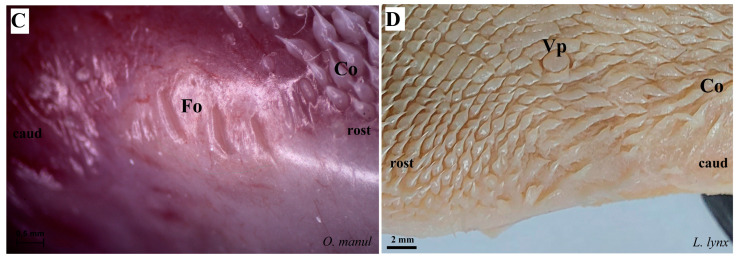
Stereoscopic view of the lateral left (**A**,**B**,**D**) and right (**C**) lingual surface between the body and root of the tongue in four species of undomesticated Felidae: *N. nebulosa* (**A**), *P. leo bleyenberghi* (**B**), *O. manul* (**C**) and *L. lynx* (**D**). (**A**) Several smooth folds separated by parallel grooves within the foliate papillae area. (**B**) Lack of the foliate papillae. (**C**) Smooth folds separated by five grooves, which form the foliate papillae. (**D**) Lack of the foliate papillae. Bar = 2 mm (**B**,**D**); bar = 0.5 mm (**A**,**C**). Abbreviations: caud—caudal orientation of the mechanical lingual papillae, Co—conical papilla, Fo—foliate papilla, rostr—rostral orientation of the mechanical lingual papilla, Vp—vallate papilla.

The dorsal surface of the apex and body of the tongue was covered with lingual papillae, the most numerous of which were mechanical filiform papillae (Figure 2A–D), varying in size as Fi_I_, Fi_II_, Fi_III_, Fi_IV_ and Fi_V_ (Figure 2A–D, Figure 3A–D and Figure 4A–G, and Table 3) subtypes. Filiform papillae Fi_I_—Fi_V_ also differed in their location on the dorsal surface of the tongue. Filiform papillae subtype I (Fi_I_) was directed caudally and present on the apex of the tongue (Figure 2A–D and Figure 4A,C). Filiform papillae subtype II (Fi_II_) was the largest subtype of the filiform papillae; it also had a caudal orientation and was present on the rostral part of the body of the tongue just behind the lingual apex (Figure 3A–D). Filiform papillae subtype III (Fi_III_) had a medio-caudal orientation and was present on the lateral parts of the body of the tongue (Figure 2A–D). Filiform papillae subtype IV (Fi_IV_) had a caudal orientation located on the central part of the body of the tongue between Fi_III_ papillae on each lateral side (Figure 2A–D). Filiform papillae subtype V (Fi_V_) had a caudal orientation and was located between the body and the root of the tongue (Figure 2A–D). Additionally, mechanical conical papillae (Co) were present on the dorsal surface of the root of the tongue in all four species (Figure 2A–D). Small filiform papillae were also identified, between which there were unevenly visible fungiform papillae (Figure 2A–D and Figure 4A). The gustatory lingual papillae were fungiform lingual papillae, vallate papillae and foliate papillae (Figure 2A–D, Figure 4A, Figure 5A–D and Figure 6A–D). Fungiform papillae were mainly present in four Felidae species on the dorsal lingual surface of the apex and body of the tongue, and some were between vallate papillae on the root of the tongue. Vallate papillae were arranged in a variable number of each species and were in a V-shape between the body and the root of the tongue (Figure 2A–D and Figure 5A–D). The number of vallate papillae in the four analyzed species is presented in Table 2. The shape of the vallate papillae varied from round to elongated, and some vallate papillae were subdivided with an irregular surface, as shown macroscopically (Figure 5A–D). Another group of gustatory papillae was foliate papillae, and *O. manul* has the best visible smooth folds separated by parallel grooves (Figure 6C). Foliate papillae were also observed in *N. nebulosa* (Figure 6A), while foliate papillae were absent in *P. leo bleyenberghi* and *L. lynx* (Figure 6B,D). The number of folds separated by grooves of foliate papillae was five and six on both lateral sides of the root of the tongue (Figure 6C) in *O. manul*.

**Figure 7:**
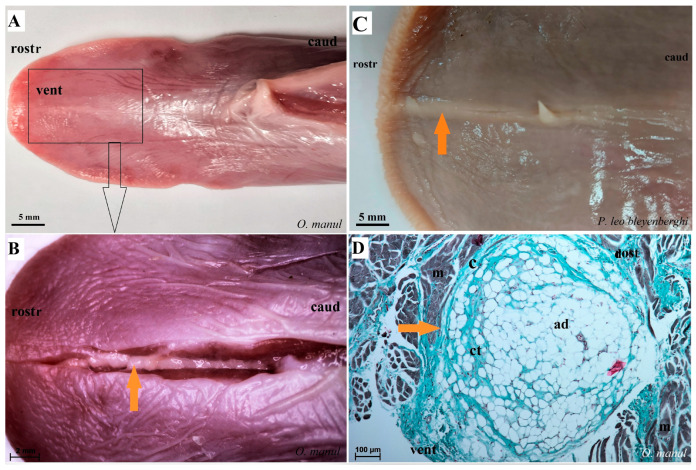

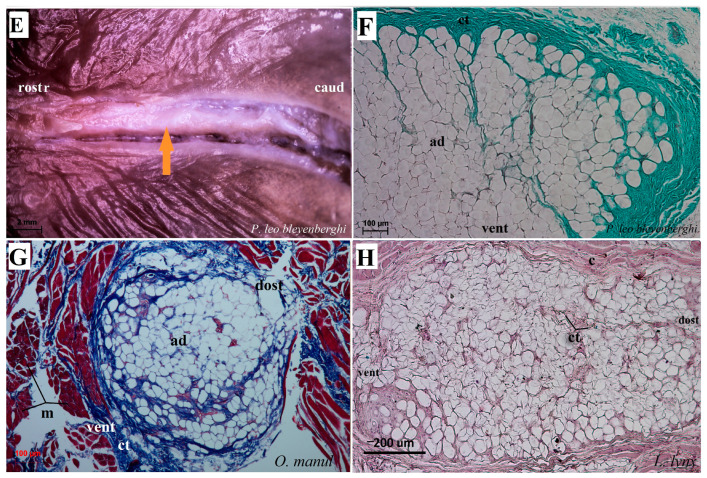
Macroscopic view (**A**–**C**,**E**) and histological examination (**D**,**F**–**H**) of the lyssa of *O. manul* (**A**,**B**,**D**,**G**), *P. leo bleyenberghi* (**C**,**E**,**F**) and *L. lynx* (H). (**A**,**C**) Ventral smooth surface of the tongue with lyssa (orange arrow). (**B**) Magnification (black rectangle) of the ventral surface of the tongue with well-defined lyssa. (**D**) Transverse section of the lyssa. (**E**) Well-defined lyssa, which is elongated in shape. (**F**) Transverse section of the lyssa; Masson–Goldner trichrome staining. (**G**) Transverse section of the lyssa; Azan trichrome staining. (**H**) Transverse section of lyssa; H&E staining. Bar = 5 mm (**A**,**C**); bar = 2 mm (**B**,**E**); bar = 100 µm (**D**,**F**,**G**), bar = 200 µm (**H**). Abbreviations: ad—adipocytes of the lyssa, c—capsule of the lyssa, caud—caudal orientation, ct—connective tissue, m—muscle fibers, dost—dorsal orientation, rostr—rostral orientation, vent—ventral surface of the tongue.

The ventral lingual surface was smooth and had no lingual papillae (Figure 4B,D and Figure 7A–C). However, within the median surface of the apex of the tongue, lyssa was visible (Figure 7A–C,E). Macroscopically, the lyssa was rather white in all four studied species, and, at the same time, it was developed to different degrees in individual Felidae, but the thinnest and least developed one was in *P. leo bleyenberghi*, where the lyssa was about 3 cm long in the male and 2 cm long in the female (Table 2). In the cross-section, the lyssa was oval in shape, and its caudal part was thinner than the rostral and middle parts (Figure 7A–C,E).

### 3.2. Light Microscopic Comparative Analyses

**Figure 8:**
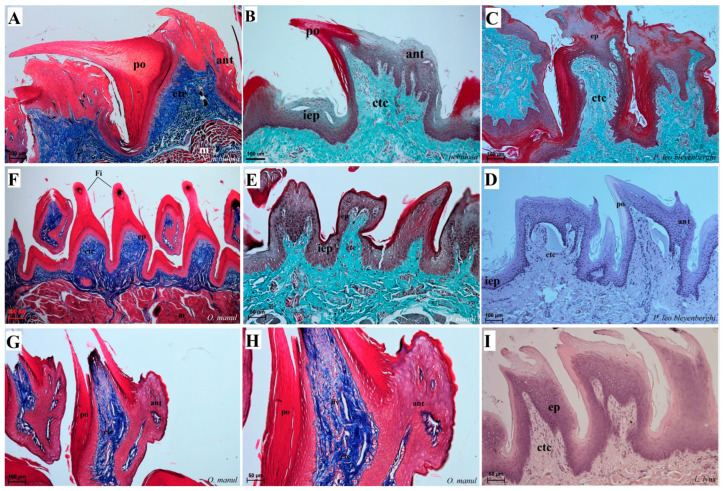
Histological structure of the filiform papillae of the tongue of the four undomesticated species of Felidae: *N. nebulosa* (**A**,**B**), *P. leo bleyenberghi* (**C**,**D**), *O. manul* (**E**–**H**) and *L. lynx* (I). (**A**) Longitudinal section of the filiform papillae with well-defined stratum granulosum and stratum corneum of the papillary stratified squamous epithelium. (**B**) Longitudinal section of the filiform papillae with interpapillary epithelium. (**C**,**F**) Rostral view of the filiform papillae. (**D**,**E**–**I**) Longitudinal section of the filiform papillae with the enlarged stratum granulosum. Azan trichrome staining (**A**,**F**,**G**,**H**); Masson–Goldner trichrome staining (**B**,**C**,**E**); H&E staining (**D**,**I**). Bar = 200 µm (**A**,**F**); bar = 100 µm (**B**,**C**,**D**,**G**); bar = 50 µm (**E**,**H**,**I**). Abbreviations: ant—anterior part of the filiform papillae (prominent stratum granulosum), ctc—connective tissue core, ep—epithelium, Fi—filiform papilla, iep—interpapillary epithelium, m—muscle fibers, po—posterior part of filiform papillae (prominent stratum corneum).

**Figure 9:**
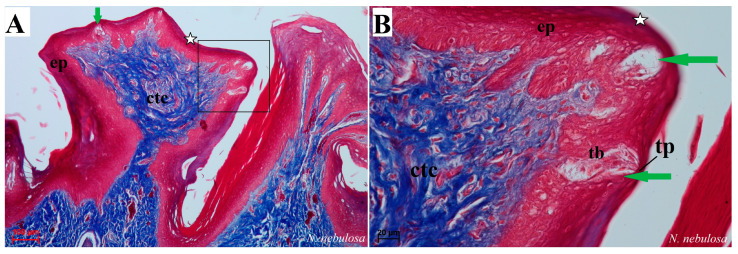

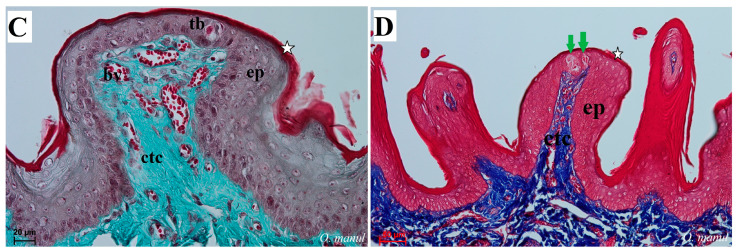
Histological picture of the fungiform papillae of the tongue of undomesticated species of Felidae: *N. nebulosa* (**A**,**B**) and *O. manul* (**C**,**D**). (**A**) Longitudinal section of the fungiform papilla with several taste buds within the epithelium. (**B**) Magnification of the fungiform papillae with two elongated-shaped taste buds (green arrow). Thin keratinized layer is visible (white asterisk). (**C**) Longitudinal section of dome-shaped fungiform papilla with numerous blood vessels within the connective tissue core of this papilla. Single taste bud present within the epithelium as well as a thin keratinized layer (white asterisk). (**D**) More elongated fungiform papillae with two taste buds. Azan trichrome staining (**A**,**B**,**D**); Masson–Goldner trichrome (**C**). Bar = 100 µm (**A**); bar = 50 µm (**D**); bar = 20 µm (**B**,**C**). Abbreviations: bv—blood vessels, ctc—connective tissue core, ep—epithelium, tb—taste bud, tp—taste pore.

**Figure 10:**
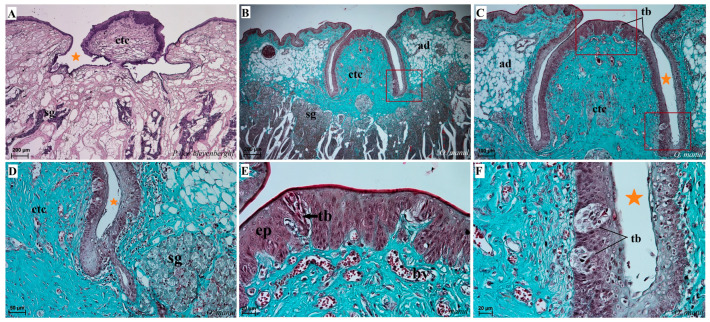
Histological pictures of the vallate papillae of the tongue of *P. leo bleyenberghi* (**A**) and *O. manul* (**B**–**F**). (**A**–**C**) Longitudinal section of the vallate papilla with papillary groove (orange asterisk). (**D**,**F**) Magnification of the lateral epithelium of the papillae with several taste buds. (**E**) Magnification of the dorsal epithelium of the vallate papilla. H&E staining (**A**); Masson–Goldner trichrome staining (**B**–**F**). Bar = 200 µm (**A**,**B**); bar = 100 µm (**C**); bar = 50 µm (**D**); bar = 20 µm (**E**,**F**). Abbreviations: ad—adipocytes, bv—blood vessels, ctc—connective tissue core, ep—epithelium, m—muscle fibers, sg—serous glands, tb—taste bud.

**Figure 11:**
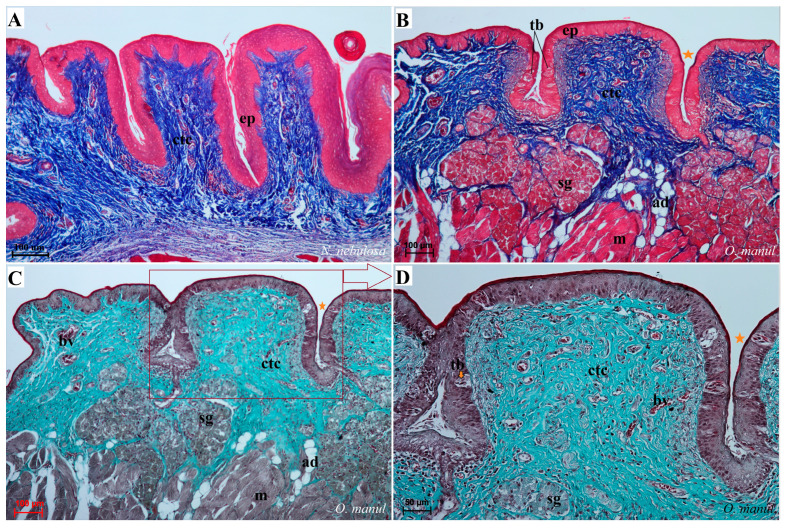
Histological pictures of the foliate papillae area of the tongue of *N. nebulosa* (**A**) and *O. manul* (**B**–**D**). (**A**) Lack of taste buds within the lateral side of the tongue within the epithelium of the foliate papillae. (**B**,**C**) Foliate papillae with several well-visible elongated taste buds beneath the papillae serous lingual glands are present. Deep sulcus of the foliate papilla (orange asterisk). (**D**) Magnification of the foliate papilla fold with several taste buds (orange arrow). Azan trichrome staining (**A**,**B**); Masson–Goldner trichrome staining (**C**,**D**). Bar = 100 µm (**A**–**C**); bar = 50 µm (**D**). Abbreviations: ad—adipocytes, bv—blood vessels, ctc—connective tissue core, ep—epithelium, m—muscle fibers, sg—serous glands, tb—taste bud.

Histological analysis showed that different subtypes of filiform papillae were composed of connective tissue core and the overlying stratified squamous epithelium, with keratinized layers to varying degrees (Figure 8). A distinct stratum granulosum on the anterior side of each subtype II filiform papillae and a distinct stratum corneum of the stratified squamous epithelium on the posterior side of each subtype II filiform papillae were observed in the longitudinal section (Figure 8A,B,D,G,I). Fungiform papillae had a distinct connective tissue core, while their outer surface was composed of multilayered squamous epithelium, with a delicate keratinized layer (Figure 9). A few elongated taste buds were located predominantly in the apical parts of the fungiform papillae (Figure 9A,C,D) and some on the lateral side of these papillae (Figure 9B). In *N. nebulosa*, the shape of the surface of fungiform papillae was more irregular in the dorsal part (Figure 9A,B) in contrast to the *O. manul* in which the surface of these fungiform papillae was smooth (Figure 9C,D). The vallate papillae were surrounded by a deep papillary groove (Figure 10A–C), especially in *O. manul* (Figure 10B,C). Numerous taste buds were observed from within the epithelium forming vallate papillae not only in its lateral walls but also within its dorsal epithelium (Figure 10B,C,E,F). The taste buds were elongated (Figure 10E,F). Grooves and folds within the foliate papillae area in *N. nebulosa* were characterized by a lack of taste buds in the epithelium in all slices (Figure 11A). In contrast, the foliate papillae in *O. manul* were distinct, and the sidewalls of the individual folds of foliate papillae contained taste buds (Figure 11B–D).

The lyssa structure in four species was dominated by adipose tissue (Figure 7D,F,G,H). Numerous adipocytes forming the main stroma of lyssa were surrounded by connective tissue capsules, as well as connective tissue fibers penetrating deep into it between the adipocytes (Figure 7D,F). The lyssa was also surrounded by a layer of striated muscle fibers.

**Figure 12:**
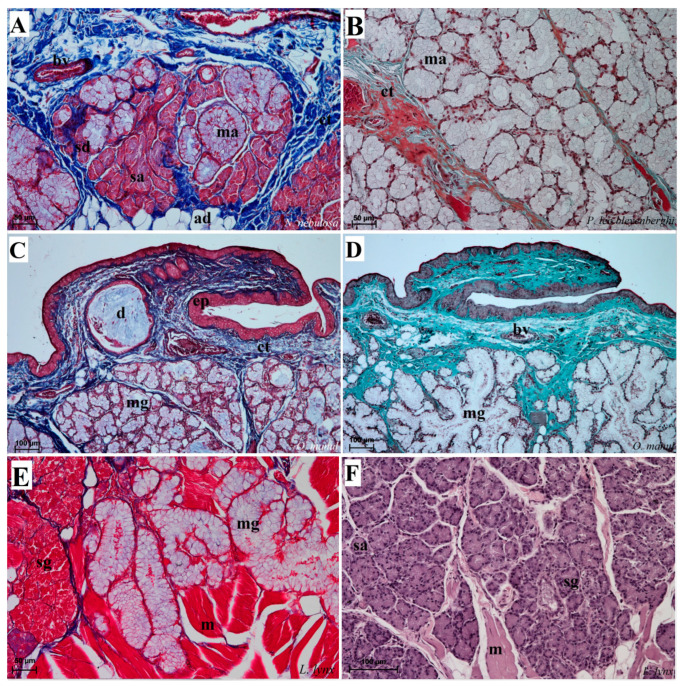
Histological pictures of the lingual glands of the root of the tongue in four species of undomesticated Felidae: *N. nebulosa* (**A**), *P. leo bleyenberghi* (**B**), *O. manul* (**C**,**D**) and *L. lynx* (**E**,**F**). (**A**) Mucoserous glands with presence of mucous and serous acini. Some mucous acini with serous demilunes are visible. (**B**) Serous glands beneath vallate papilla. (**C**) Longitudinal section of the mechanical lingual papilla and cross-section of the duct of the lingual glands as well as mucoserous glands with the dominance of mucous acini. (**D**) Mucoserous glands with dominance of mucous acini. (**E**) Mucoserous glands of the root of the tongue. (**F**) Serous glands in the tongue root. Azan trichrome staining (**A**,**C**,**E**); Masson–Goldner trichrome (**B**,**D**); H&E staining (**E**). Bar = 100 µm (**C**,**D**,**F**); bar = 50 µm (**A**,**B**,**E**). Abbreviations: ad—adipocytes, bv—blood vessel, ep—epithelium, ct—connective tissue, m—muscle fibers, ma—mucous acini, mg—mucoserous glands with dominant mucous acini (ma), sa—serous acini, sg—serous glands, sd—serous demilunes.

**Figure 13:**
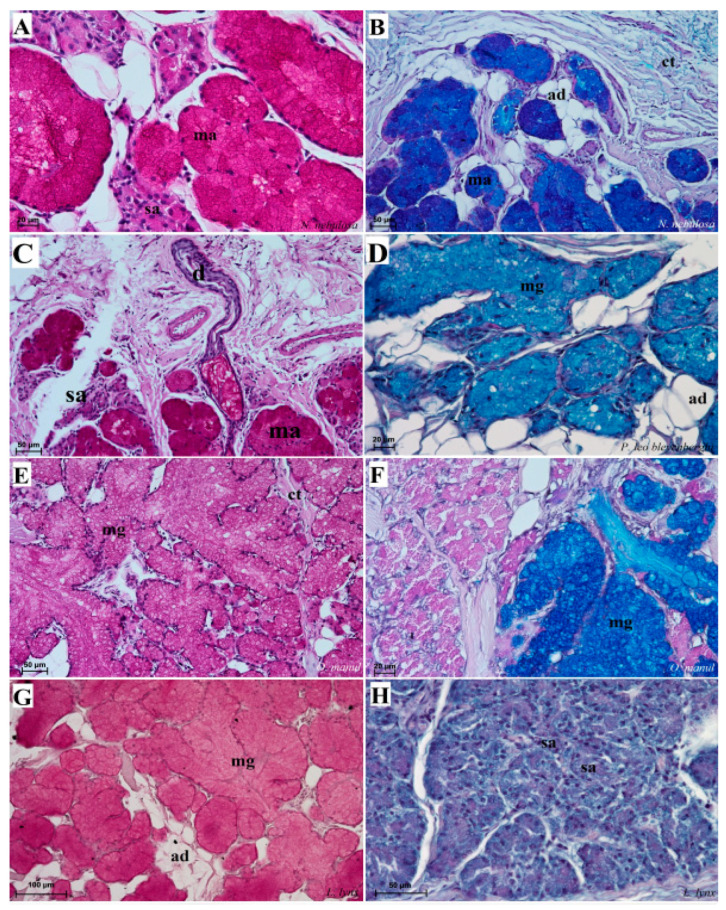
Histochemical analysis (PAS staining, PAS-AB pH2.5 staining) of the mucous synthesis in the posterior lingual glands of the four species of undomesticated Felidae: *N. nebulosa* (**A**,**B**), *P. leo bleyenberghi* (**C**,**D**), *O. manul* (**E**,**F**) and *L. lynx* (G,H). (**A**) Strong positive reaction (+++) in mucous acini (magenta) and weakly positive reaction (+) in serous acini. (**B**) Strong positive reaction (+++) in mucous acini (dark blue). (**C**) Positive reaction (++) in mucous acini (magenta). (**D**) Positive reaction (++) (blue) in mucous acini. (**E**) Positive reaction (++) in mucous acini (light magenta). (**F**) Strong positive reaction (+++) (dark blue) in mucous acini and negative reaction (−) in serous acini. (**G**) Weakly positive reaction (+) (light magenta) in mucous acini. (**H**) Negative reaction (−) in serous acini. PAS staining (**A**,**C**,**E**,**G**); PAS-AB pH2.5 staining (**B**,**D**,**F**,**H**). Bar = 100 µm (**G**); bar = 50 µm (**B**–**E**,**H**); bar = 20 µm (**A**,**F**). Abbreviations: ct—connective tissue, m—muscle fibers, ma—mucous acini, mg—mucoserous glands with the dominance of mucous acini, sa—serous acini, sg—serous glands.

**Figure 14:**
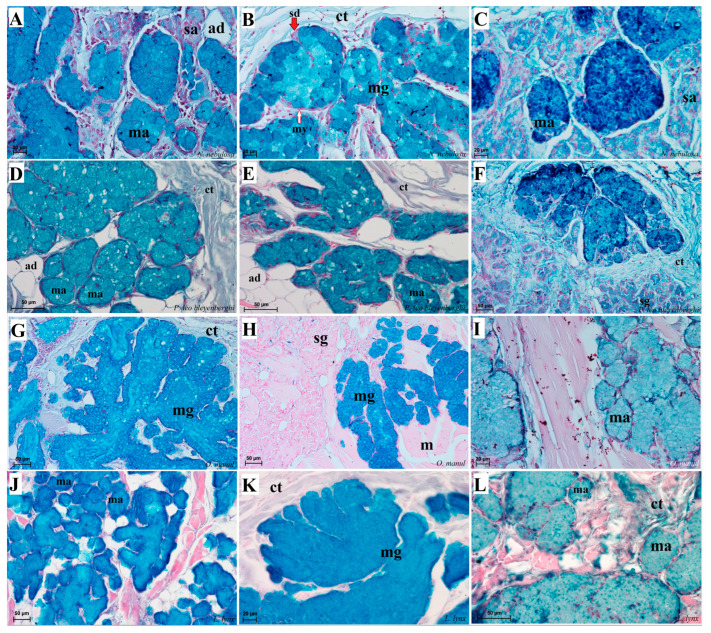
Histochemical analysis (AB pH 1.0, AB, pH 2.5 and HDI staining) of the mucous synthesis in the posterior lingual glands of the four species of undomesticated Felidae: *N. nebulosa* (**A**–**C**), *P. leo bleyenberghi* (**D**–**F**), *O. manul* (**G**–**I**) and *L. lynx* (**J**–**L**). (**A**,**D**,**G**,**J**) Mucoserous glands (with dominance of mucous acini). Strong positive reaction (+++) in mucous acini. (**B**,**E**,**H**,**K**) Strong positive reaction (+++)—blue color in mucous acini, while negative reaction in serous cells of serous demilunes (**B**). (**C**,**F**,**I**,**L**) Strong positive reaction (+++) in mucous acini (**C**,**F**), positive reaction (+) in mucous acini (**I**,**L**) and negative reaction (−) in serous acini (**C**,**F**). AB pH2.5 staining (**A**,**D**,**G**,**J**); AB pH1.0 staining (**B**,**E**,**H**,**K**); HDI staining (**C**,**F**,**I**,**L**). Bar = 50 µm (**D**–**H**,**J**,**L**); bar = 20 µm (**A**–**C**,**I**,**K**). Abbreviations: ct—connective tissue, m—muscle fibers, ma—mucous acini (round secretory units), mg—mucoserous glands with the dominance of mucous acini, my—myoepithelial cell (white arrow), sa—serous acini, sd—serous demilunes (red arrow), sg—serous glands.

Numerous mixed mucoserous glands were present in the root of the tongue area in all four Felidae species (Figure 12). Serous glands were located under the vallate and foliate papillae, and their mouths were visible towards the base of the groove of these papillae (Figure 10A,B,D and Figure 11B–D). Histochemical analysis of posterior lingual glands showed differences between the Felidae (Figure 13 and Figure 14). The strongest PAS response was observed in the mucous acini of the lingual glands in *N. nebulosa* (Figure 13A), while the weakest PAS response was seen in the mucous acini of *O. manul* (Figure 13E). This proves the dominance in *N. nebulosa* of neutral glycoconjugates in the mucous acini of the lingual glands. The PAS-AB pH2.5 staining showed a strong positive reaction (dark blue) in mucous acini (Figure 13B) and a negative reaction in serous acini (rounded secretory units) (Figure 13D,F,H), which confirms the presence of secretion containing combinations of both acidic and neutral glycoconjugates. The AB pH1.0 and AB pH2.5 staining showed positive reactions in mucous acini in all four species (Figure 14A,B,D,G,H,J,K), as evidenced by the presence of sulfated glycoconjugates. The HDI staining showed a strong positive reaction in mucous acini in *N. nebulosa* (Figure 14C) and *P. leo bleyenberghi* (Figure 14F), which confirms the presence of carboxylated mucopolysaccharides or sulfated mucopolysaccharides (Figure 14C,F,I,L).

**Figure 15:**
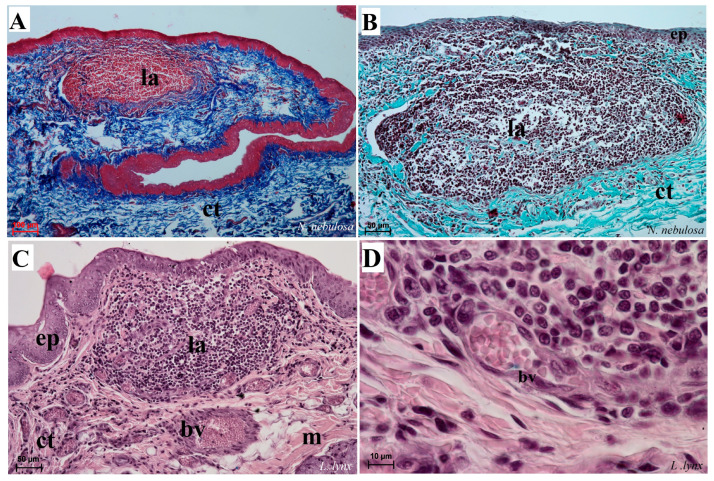
Histological analysis of the dorsal surface of root of the tongue with the aggregation of the lymphocytes and macrophages of *N. nebulosa* (**A**,**B**) and *L. lynx* (**C**,**D**). (**A**–**C**) Aggregation of lymphocytes within the root of the tongue. (**D**) Magnification of the venule containing numerous erythrocytes surrounded by aggregates of the leukocytes (mainly macrophages and lymphocytes). Azan trichrome staining (**A**); Masson–Goldner trichrome staining (**B**); H&E staining (**C**,**D**). Bar = 100 µm (**A**–**C**); bar = 50 µm (**D**). Abbreviations: bv—blood vessel, ct—connective tissue, ep—epithelium, la—aggregation of the lymphoid tissue, m—muscle fibers.

Aggregation of the lymphocytes and macrophages was found within the root of the tongue directly under the squamous epithelium, especially in *N. nebulosa* (Figure 15A,B) as well as in *L. lynx* (Figure 15C,D). Numerous blood vessels were also visible within the aggregation dominated by macrophages and monocytes.

### 3.3. Scanning Electron Microscopic (SEM) Comparative Analyses

**Figure 16:**
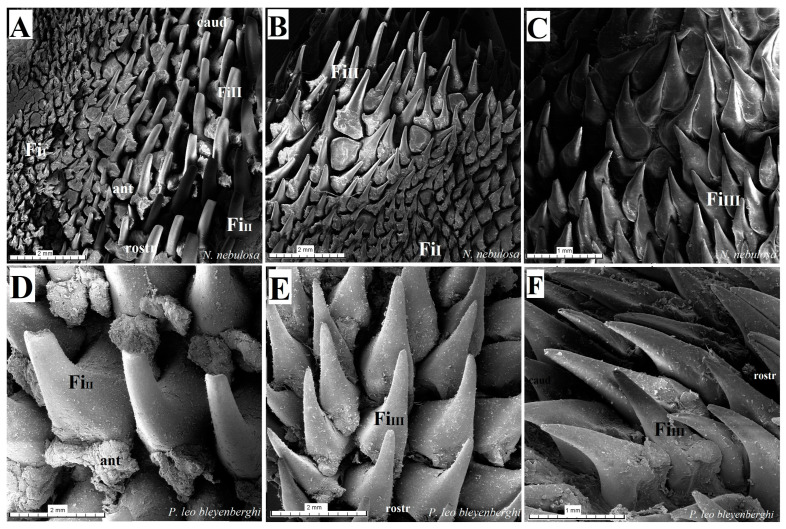

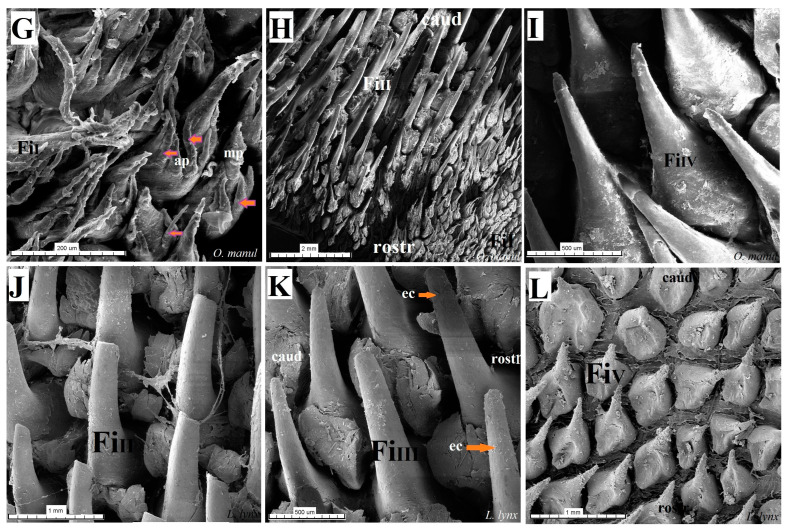
Scanning electron microscopic (SEM) pictures of the filiform papillae of the tongue of the four undomesticated species of Felidae: *N. nebulosa* (**A**–**C**), *P. leo bleyenberghi* (**D**–**F**), *O. manul* (**G**–**I**) and *L. lynx* (**J**–**L**). (**A**,**B**) Filiform papillae subtype I and II between the apex and body of the tongue. (**C**) Filiform papillae subtype III from lateral part of the body of the tongue. (**D**) Largest filiform papillae subtype II with the best-developed anterior part of each papilla. (**E**,**F**,**I**,**K**) Filiform papillae subtype III without additional projections. (**G**) Small filiform papillae subtype I with main base of papillae and several additional projections on the anterior part of main papilla. (**H**) High filiform papillae subtype II between the apex and body of the tongue. (**J**) Largest filiform papillae subtype II. (**L**) Short filiform papillae subtype V between body and root of the tongue. Bar = 2 mm (**A**,**B**,**D**,**E**,**H**); bar = 1 mm (**C**,**F**,**J**,**L**); bar = 500 µm (**I**,**K**); bar = 200 µm (**G**). Abbreviations: ant—anterior part of the mechanical lingual papillae, ap—additional projection (purple arrows), caud—caudal orientation of the papillae, ec—exfoliated cell (orange arrow), Fi_I_—filiform papillae subtype I, Fi_II_ —filiform papillae subtype II, Fi_III_—filiform papillae subtype III, Fi_IV_—filiform papillae subtype V, Fi_V_ —filiform papillae subtype V, mp—main part of the lingual mechanical papilla subtype Fi_I_, rostr—rostral orientation of the papillae.

SEM analysis of the dorsal surface of the tongue in four individual Felidae species allowed for the differentiation of mechanical filiform lingual papillae into five subtypes based on their shape (Figure 16). Filiform papillae type I (Fi_I_) was present on the lingual apex of the four examined species, and it was formed by the main papilla and several additional projections (Figure 16A,G). Type I of filiform papillae was the smallest in all felids; in *O. manul*, this Fi_I_ had several (less than eight) additional small elongate projections (Figure 16G). Similar additional projections (less than 10) were recognized in *L. lynx* and *N. nebulosa*. The tips of Fi_I_ papillae were conical in all four felids. Filiform papillae type II (Fi_II_) had a spatulate tip, and the anterior base of each main papilla was enlarged in all four species (Figure 16A,D,H,J). The additional projections of Fi_II_ were not observed in comparison to Fi_I_. The thickened area at the base of Fi_II_ papillae was typical in all examined species, with the biggest in *P. leo bleyenberghi* (Figure 16D). Filiform papillae type III (Fi_III_) had pointed tips, and they look more conical in shape (Figure 16C,E,F,K). The thickened area at the base of these Fi_III_ papillae was observed in *P. leo bleyenberghi* (Figure 16E) as well as in *L. lynx* (Figure 16K), while in *N. nebulosa*, it was smooth at the base (Figure 16C). Filiform papillae type IV (Fi_IV_) were conically elongated with pointed apexes (Figure 16I) in all four species without additional projections, while Fi_V_ had a broad base (Figure 16L) and was significantly shorter than Fi_II_, Fi_III_ and Fi_IV_ (Figure 16L). On the surface of filiform papillae, numerous exfoliated cells were visible (Figure 16K). Additionally, the long conical papillae were observed on the dorsal surface of the root of the tongue in all examined felids.

**Figure 17:**
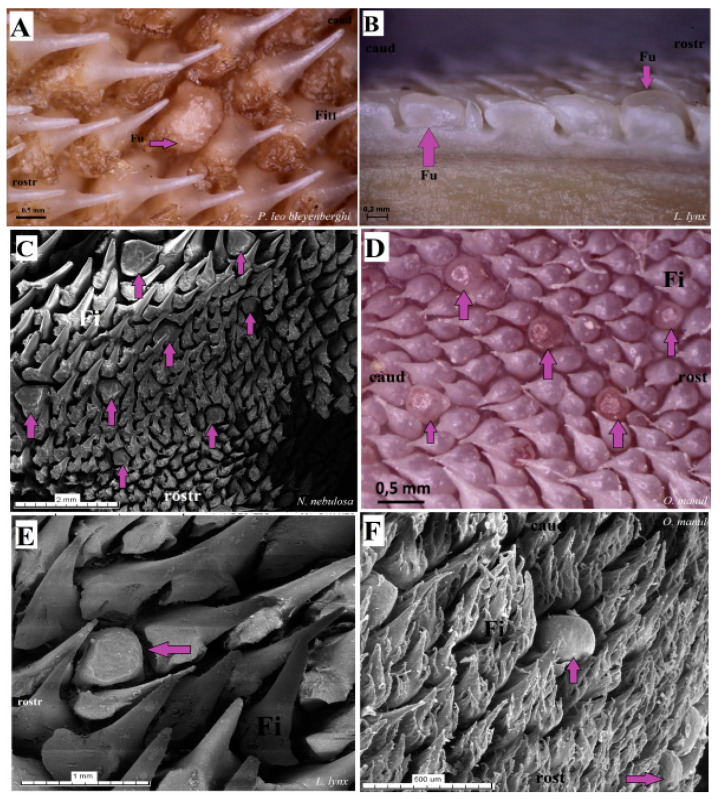
Macroscopic view (**A**,**B**,**D**) and scanning electron microscopy (SEM) (**C**,**E**,**F**) pictures of the fungiform papillae of the undomesticated species of Felidae. (**A**) Dorsal view of the one dome-shaped fungiform papilla (purple arrow) of the *P. leo bleyenberghi* with several filiform papillae. (**B**) Longitudinal section of the body of the tongue of *L. lynx* with well-visible sunken-shaped fungiform papillae between filiform papillae. (**C**) Eight sunken-shaped fungiform papillae within the filiform papillae on the dorsal surface of the body of tongue of *N. nebulosa*. (**D**) Five round-shaped fungiform papillae of the tongue of *O. manul*. (**E**) SEM picture of the one sunken-shaped fungiform papillae of the tongue of *L. lynx*. (**F**) Structure of the two smooth dome-shaped fungiform papillae of *O. manul* SEM. Bar = 2 mm (**C**); bar = 1 mm (**E**); bar = 0.5 mm (**A**,**D**,**F**); bar = 0.2 mm (**B**). Abbreviations: caud—caudal orientation of the filiform papillae, Fi—filiform papilla, Fu—fungiform papilla (purple arrow), rostr—rostral orientation of the filiform papillae.

**Figure 18:**
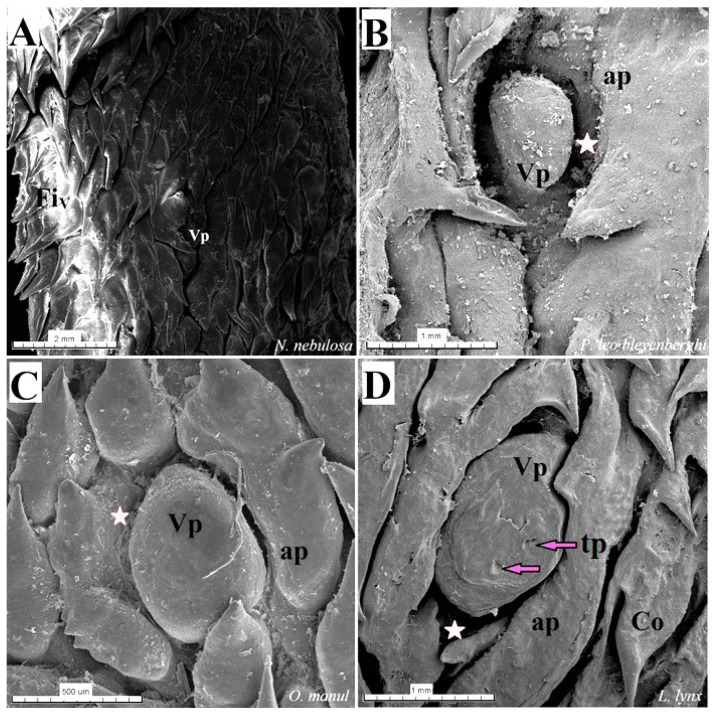
Scanning electron microscopic (SEM) pictures of the vallate papillae of the tongue of the four species of undomesticated Felidae: *N. nebulosa* (**A**), *P. leo bleyenberghi* (**B**), *O. manul* (**C**) and *L. lynx* (**D**). (**A**) An elongated-shaped vallate papilla. (**B**) Well-visible deep papillary groove and oval- shaped vallate papilla. (**C**) Vallate papillae encircled by an irregular annular pad. (**D**) Two taste pores on the dorsal surface of the vallate papilla. Bar = 2 mm (**A**); bar = 1 mm (**B**,**D**); bar = 500 (**C**). Abbreviations: ap—annular pad with irregular surface with exfoliated cells, Co—conical papilla, tp—taste pore of the taste bud (purple arrow), Vp—vallate papilla, * (asterisk)—groove of the vallate papilla.

**Figure 19:**
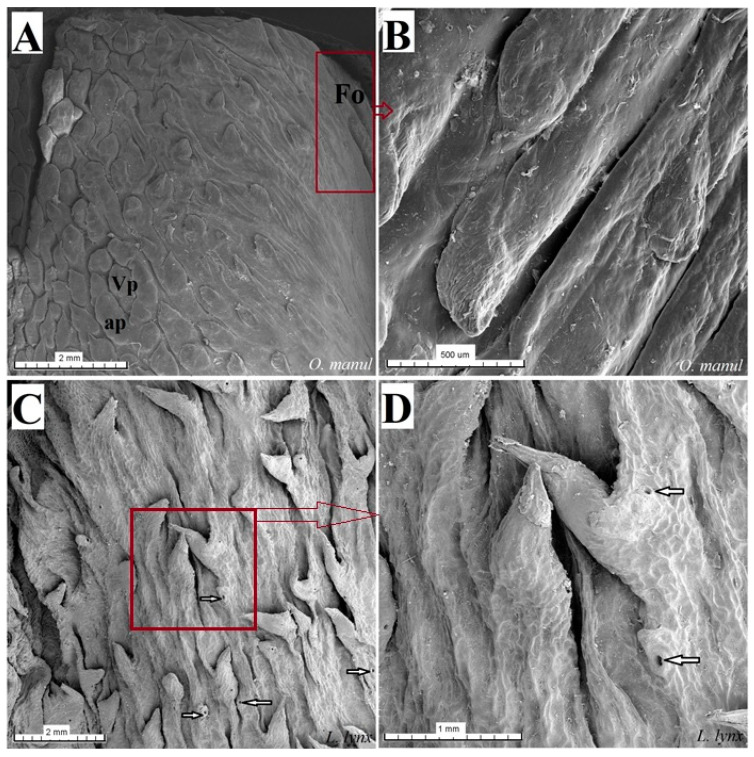
Scanning electron microscopy (SEM) pictures of the foliate papillae area of the tongue of *O. manul* (**A**,**B**) and lateral area of the tongue between the body and root of the *L. lynx* (**C**,**D**). (**A**) Foliate papillae area on the right part of the sample (maroon rectangle) and vallate papillae surrounded by the annular pad. (**B**) Magnification of the folds and grooves which form the foliate papilla. (**C**) Several round openings (white arrows) of the glandular ducts of posterior lingual glands on the lateral posterior surface of the tongue and several well-visible conical papillae. (**D**) Magnification of the opening of the lingual gland ducts and two conical lingual papillae. Bar = 2 mm (**A**,**C**); bar = 1 mm (**D**); bar = 500 µm (**B**). Abbreviations: ap—annular pad, Fo—foliate papilla, Vp—vallate papilla.

The SEM study of gustatory papillae showed the presence of their irregular surface depending on the type of papillae and the presence of taste pores of the taste buds on the dorsal surface of fungiform papillae and vallate papillae (Figure 17C,E,F and Figure 18D). Fungiform papillae were dome-like in shape or blunt-shaped in all four analyzed species (Figure 17). The number of taste pores on the surface of the fungiform papillae was no more than four–five depending on the species. The vallum of the vallate papillae was surrounded by a deep groove and annular pad (Figure 18). The widest groove around the vallate papillae was found in *P. leo bleyenberghi* (Figure 18B). The annular pad shape was different in each species. The irregular annular pad was most typical for *O. manul* (Figure 18C), while it was rather smooth for *L. lynx* (Figure 18D). An elongated shape of the annular pad was observed in the *P. leo bleyenberghi* and *N. nebulosa* (Figure 18A,B). The widest annular groove was visible in *P. leo bleyenberghi* (Figure 18B). The area of the foliate papillae was characterized by folds and parallel grooves in *O. manul* (Figure 19A,B), while in the case of *P. leo bleyenberghi* and *L. lynx*, this area lacked foliate papillae. Round openings of posterior lingual glands were present in the vicinity of the conical papillae between the body and the root of the tongue in *L. lynx* (Figure 19C,D), which was similar to the other three species of felids.

## 4. Discussion

Wild felids are representatives of mammals dominated by hypercarnivorous diets, hence the anatomical structure of the entire digestive system, which is typical for these species and enables proper food processing. The species of felids from which the tongues were taken for this study, although they are closely related (as representatives of one Felidae family within the Feliformia order), live in different areas in the natural environment; therefore, the basis of their diets also differs (*N. nebulosa* is found in the terranes of central and southern Asia, *P. leo bleyenberghi* lives in nature in arid areas in Angola and Zimbabwe, *O. manul* lives in extremely dry areas where some of the lowest temperatures on the globe are recorded, falling even to −50 °C, while *L. lynx* is a species that lives mainly in central and northern Europe and central Asia [8,34]. One of the typical features of the studied representatives of undomesticated Felidae, including *P. leo bleyenberghi*, *L. lynx* and *O. manul*, is long canines, which are exceptionally long in *N. nebulosa*, which makes it easier for these species of predatory mammals to obtain food. An oily adaptive feature, in addition to the specialized structure of the dentition, maxilla and mandible, is also the characteristic structure of their tongue, differing in the microstructure of its surface in the neonatal period compared to its structure in young and adult individuals [12,19,22,35]. The unique microstructure of the surface of the tongue of felids, as well as the system of other muscles of the tongue, allows the use of it for the laceration of meat, as well as retaining food within the mouth and the frequent cleaning of fur [7,8,16,36].

### 4.1. Papillae of the Tongue

Filiform papillae, due to their characteristic shape, have been divided into five subtypes in *N. nebulosa*, *P. leo bleyenberghi*, *L. lynx* and *O. manul*. The comparable features of filiform papillae were also observed in other felids, in which such analyzes have so far been carried out in the subfamily *Pantherinae* [9,13,15,18] and the subfamily *Felinae* [5,6,16,20,21,22]. In the case of *Panthera onca* [15], *Panthera pardus* [9] and *Panthera pardus saxicolor* [18], some filiform papillae were described as cylindrical in shape, while some filiform papillae contain secondary projections, which is similar to the four species in this study, where filiform papillae from the apex had distinct secondary projections. Furthermore, in *Panthera tigris tigris*, additional projections in the construction of filiform papillae were identified [17]. Similarities in the mechanical lingual papillae microstructures within the *Pantherinae* and *Felinae* subfamilies also confirm their comparable role in food processing as well as in the behavior of these species, including the cleaning of fur [36]. When pieces of food are scraped off the bone, mechanical papillae facilitate the movement of these pieces towards the throat, especially type II of the filiform papillae due mostly to their size and backward orientation. Mechanical lingual papillae in the domestic cat were determined according to Boshell et al. [21] as short Fi on the tip, with several conical processes from the base of each papilla, and a large mound with a single sharp spinous process projecting Fi posteriorly on the midportion of the tongue, whereas other authors described these papillae as small (short tubular process), large (one main process and several small processes) and middle-sized Fi and the presence of giant conical papillae [20]. It is interesting that the mechanical papillae of felids feature a distinctive cavity at the tip of these papillae, which is a support for wicks saliva from the oral cavity [36] and is comparable to the microstructure of the filiform papillae in all the species examined in our study. Ojima et al. [23,24] distinguished five types of filiform papillae in Japanese cats, where I–III contain the main process, which has a large spoon-shaped and concave network process, while the other two types contain only the main process. This division is comparable to that described in the present work on four species of undomesticated Felidae; however, the shape and presence of additional projections in *N. nebulosa*, *P. leo bleyenberghi*, *O. manul* and *L*. *Lynx* differed between them and Japanese cats. Such a varied distribution of particular types of filiform papillae in the Pantherinae and Felinae subfamilies is an adaptive feature of this organ to function in a specific environment. The results obtained both by many other researchers and in our observations confirm the adaptation not only of the maxilla, mandible and teeth in grinding and crushing of food. The correct microstructure of the tongue surface, including the proper keratinization process, and lingual muscles that provide movement of the tongue also play a key role here. At the same time, the presence of conical papillae on the surface of the tongue was found in *Puma concolor* [6], *Prionailurus viverrinus* [14] and *Catopuma temminckii* [10], while in the four species we studied, conical papillae were characterized in the area of the root of the tongue.

Fungiform papillae, vallate papillae as well as foliate papillae are gustatory papillae responsible for receiving the sense of smell. This function is closely related to the presence of well-defined neuroanatomical structures—taste buds. The presence of these structures affects the degree of development of the organ of taste in animals, including felids. Many studies on the organ of taste indicate its complex structure and great diversity. Fungiform papillae are found in all representatives of the *Felinae* and *Pantherinae* subfamilies. However, their distribution on individual parts of the dorsal surface of the tongue differed between species and individuals [5]. In cats, the presence of two–three taste buds on the dorsal epithelium of fungiform papillae was observed in adult animals [22], so the presence of taste buds was typical, similar to all four species of *N. nebulosa*, *P. leo bleyenberghi*, *O. manul* and *L. lynx*. However, Robinson and Winkles [37] obtained different results in their study on the fungiform papillae in cats, where the mean number of taste buds was even 16.6. The difference was noted in the number of taste buds on the surface of these papillae and in the shape between the *Pantherinae* and *Felinae* subfamilies. The main similarity was in their placement, as they were most numerously observed on the apex of the tongue. For further analysis of the density of fungiform papillae and their taste buds, more samples of felids tongue are necessary.

Vallate papillae differed slightly in shape but mainly in number in different species, similar to our research. A characteristic feature of cats [22] was their arrangement in a V-shape, which is also a feature comparable to other felids, including those whose tongue microstructure was analyzed in the current research, i.e., *N. nebulosa*, *P. leo bleyenberghi*, *O. manul* and *L. lynx*. The surface of vallate papillae in cats was irregular [22], which is also comparable to the appearance of these papillae in the four species we studied. A feature of vallate papilla in *Leopardus pardalis* [5] was the oval shape, i.e., similar to some of the vallate papillae in *P. leo bleyenberghi*. On the one hand, most felid vallate papillae are round in shape. However, their number in *Leopardus pardalis* was three on each side of the tongue [5], so different than in the subjects of the four species of captive felids. On the other hand, the papillae of the tongue in *Catopuma temminckii*, which were composed of primary papillae and subdivided into secondary papillae, were characterized by a different feature [10]. In addition, *Panthera tigris altaica* vallate papillae had a non-regular surface [13], similar to our analysis of four undomesticated species of Felidae. Interestingly, in *Panthera tigris tigris*, only two vallate papillae were observed by Kim et al. [17] in their research. Each of these vallate papilla was composed of two secondary papillae inside the grooves [17], which distinguishes the microstructure of this type of gustatory papillae between *Panthera tigris tigris* and species such as *N. nebulosa*, *P. leo bleyenberghi*, *O. manul* and *L. lynx*.

Foliate papillae are the rarest type of gustatory papillae in felids [9], which is also confirmed by our analysis of *P. leo bleyenberghi* and *L. lynx*. However, in *Panthera tigris tigris*, the presence of this kind of gustatory papillae was confirmed [17], while in cats, they were built with two–five lateral folds but without taste buds [22]. Furthermore, in *Panthera pardus saxicolor*, foliate papillae were present as several smooth folds separated by grooves but without taste buds [18], as in *N. nebulosa* in our observations. In the current study, however, the presence of taste buds in the foliate papillae epithelium of *O. manul* was found.

### 4.2. Lyssa

Lyssa as a structure present on the ventral side of the tongue in the middle apex of the tongue and running along the caudal direction was observed in *N. nebulosa*, *P. leo bleyenberghi*, *L. lynx* and *O. manul*, which is comparable to the observation of this structure in the domestic cat [26,38], *Leopardus pardalis* [5] and *Panthera pardus saxicolor* [18]. Moreover, the presence of lyssa in the tongue was confirmed in studies of other animal species apart from Felidae, including dogs [26,37,39], red pandas (*Ailurus fulgens fulgens*) [27] and camels [36]. Cat lyssa had a helical appearance; it was yellow in color [26] and was approximately 1.5 cm in length. Interestingly, these authors found that in the domestic cat, the pyramidal rod encircled the ventral edge of the rostral part of the lyssa [26], which was not observed in the four Felidae species in the present study. The lyssa in domestic cats was surrounded by connective tissue capsules, while its interior part was dominated by adipocytes separated by septa made of connective tissue penetrating the interior of the lyssa, which was also observed in the histological structure of the lyssa in *N. nebulosa*, *P. leo bleyenberghi*, *L. lynx* and *O. manul*. Macroscopic analysis of lyssa revealed substantial differences in the length and thickness of this structure between the analyzed undomesticated Felidae species. However, a different structure was found in *Panthera pardus saxicolor* [18], where cartilaginous tissue surrounded by a thin layer of connective tissue fibers dominated. As the function of lyssa is still unknown, we only present the assumed functions. Besoluk et al. [26] proposed that the spiral structure of feline lyssa made very quick and/or short-time lengthening of the tongue possible; therefore, the cat can effectively use its tongue for speedy lingual movements. In contrast, Shoeib et al. [38] reported that the full function of the tongue was achieved after the surgical resection of lyssa in dogs and cats. After complete healing of the tongue, all dogs and cats had a normal condition for protrusion of the tongue, prehension of foods, and liking and playing with the tongue, with no abnormal behavioral changes. Therefore, Shoeib et al. [38] proposed that the lyssa served as an elastic limb and/or skeleton of the free portion of the tongue. However, other functions were proposed by Besoluk et al. [26], who described muscle spindle-like structures in the middle third of the dog’s lyssa sheath. These structures possibly detect and/or adjust the threshold level of changes suitable for any lingual tension, thus acting as a sort of potentiometer apparatus [26]. In cats, vertical fibers of the genioglossus muscle were connected with the aboral extremity of the lyssa and possibly substituted for muscle spindles in order to regulate the lingual extending position [26]. We add one more suggestion to the previously mentioned functions. The lyssa was made of adipose tissue in all four species examined in our study. The function of adipose tissue as a shock absorber is known and commonly reported, e.g., in pads of the digital cushion [40]. We suggest that the adipose tissue of the lyssa may also serve as a shock absorber, especially during processing hard food, such as bones, in the oral cavity. Nevertheless, although the function of lyssa is still unexplained, knowledge of the structure of lyssa may be important in the event of possible tongue surgery in many species of wild felids. Moreover, differences between the male and the female in the lyssa morphology cannot be ruled out either. No study in any species was found that noted a difference in the ultrastructure of the lyssa in males and females. Besoluk et al. [26], Shoeib et al. [38] and El-Bably and Tolba [41] described the ultrastructure of the lyssa in males and females but did not describe any differences. A slight variability in the color of the adipose tissue forming the lyssa is even among domestic cats. While El-Bably and Tolba [41] reported a white lyssa, Besoluk et al. [26] described it as yellow. The composition of diets can influence the color of adipose tissue [42] so some components of a carnivorous diet could cause this slight difference in color. In our study, the lyssa was rather white in all four examined species; however, it was developed to different degrees in the individual felids. The least developed one was in *P. leo bleyenberghi* when compared in size to the length of the tongue. It is possible that with such a construction of the *P. leo bleyenberghi* tongue musculature, there is no need for a strong expansion of the lyssa, which in *N. nebulosa*, *O. manul* and *L. lynx* could play the role of a shock absorber. Further studies on the felid`s lyssa structure conducted on a much larger number of samples could be a continuation of the research mentioned above.

### 4.3. Lingual Glands

Mixed lingual glands present in the examined four species of undomesticated Felidae within the root of the tongue were characterized by the dominance of mucous acini. However, so far, little attention has been paid to the analysis of the composition of posterior lingual glands in various Felidae species. Such research was conducted, among others, on a cat [43]. According to Sozmen et al. [43], variations in staining results may be indicative of different stages in the secretory cycles if one compares the secretions of different glands, including lingual glands, because, in the four tested species of undomesticated Felidae, the positive reaction differed in severity. In addition, they may also be characterized by variability related to the sex or maturity of the individuals concerned. According to Sadeghinezhad et al. [18], the tongue glands in *Panthera pardus saxicolor* were mainly composed of serous cells and a few mucous cells, which differed significantly from the results obtained in our histological observations of the posterior lingual glands in *N. nebulosa*, *P. leo bleyenberghi*, *L. lynx* and *O. manul*. In the undomesticated captive Felidae we studied, the glands of the tongue were dominated by mucous cells, hence the glands were also referred to as mixed mucoserous glands. Nevertheless, in the connective tissue located below the vallate papillae or foliate papillae (in those which were present), serous units of these lingual glands dominated.

## 5. Conclusions

Considering the obtained results, some concordances as well as divergences between the four examined species were found based on macroscopic, histological and SEM analyses. Macroscopically, the tongues had a similar elongated shape in all four felids; the largest tongue was observed in *P. leo bleyenberghi* male, while in the female of *P. leo bleyenberghi*, the tongue was shorter and with smaller mechanical papillae. The tongues of *N. nebulosa*, *O. manul* and *L. lynx* had comparable lengths; however, they were much shorter than in *P. leo bleyenberghi*. For the further comparative study of the tongues between the males and females of different felids, a larger collection of tongues is necessary. In addition, the age of animals can influence the keratinization process of the dorsal lingual surface. Typical for the Felidae family, the presence of mechanical papillae was confirmed on the dorsal surface of the tongue (especially the typical for felids Fi type II with a spatula-tipped tip) between the apex and the rostral part of the body of the tongue in the four examined species. The keratinization level of these mechanical papillae was different between the examined species. This may be connected with the function of the tongue, especially in felids. In addition, because all the examined animals were collected from zoos, the diet of all these felids was different than in their natural environment, which can influence the level of keratinization of filiform papillae. The difference was also macroscopically and histologically confirmed in the gustatory papillae structure because, in the *P. leo bleyenberghi* and *L. lynx*, the foliate papillae were absent in contrast to *O. manul* where they were well developed. Furthermore, the type of secretion produced by the lingual glands of the root of the tongue differed slightly between the four undomesticated Felidae. The obtained results broaden the knowledge of descriptive (cognitive) anatomy and may constitute an introduction to the further analysis of the adaptation of wild felids, especially species endangered with extinction, to life in a specific environment and the consumption of food typical of these hypercarnivorous animals. In summary, a further comparative study based on a bigger size of samples should be performed for the explanation of age’s influence on the tongue structure and the possible lyssa function (including an immunohistochemical study).

## Figures and Tables

**Table 1:** Felids described in current study.

Species	Prey [8]	Habitat [8]	Collection of Research Material	Age/Sex of Animal	The IUCN Red List of Threatened Species/CITES Status
*Pantherinae*					
Clouded leopard (*Neofelis nebulosa* Griffith, 1821)	birds, monkeys, pigs, cattle, young buffalo, goats, deer and porcupines	both arboreal and terrestrial; various kinds of forests	Zoo Wrocław (Poland)	5 years, 6 months, 24 days/female	Vulnerable (VU) CITES II
Katanga lion (*Panthera leo bleyenberghi* Lönnberg, 1914)	wildebeests, impalas, other antelopes, giraffes, buffalo, wild hogs, zebras and carrions	terrestrial; grassy plains, savannahs, woodlands and scrub country	Zoo Wrocław (Poland)	13 years, 3 months, 14 days/male 14 years, 9 months, 28 days/female	Vulnerable (VU) CITES II
*Felinae*					
Eurasian lynx (*Lynx lynx* Linnaeus, 1758)	hares, small ungulates, rodents, pikas and birds	terrestrial; mostly forests	ZOO Ljubljana (Slovenia)	7 years/female	Least concern (LC) CITES II
Pallas’s cat (*Otocolobus manul* Pallas, 1776)	pikas, small rodents and ground-dwelling birds	terrestrial; steppes, deserts and rocky country	Zoo Wrocław (Poland)	4 years, 6 months, 8 days/male	Least concern (LC)

www.iucnredlist.org (accessed on 4 February 2023).

**Table 2:** Macroscopic measurements of the tongue, the number of vallate papillae and the length of lyssa in the four felids described in this study.

Species	Length (cm)	Width (cm)			Thickness (cm)			Number of Vallate Papillae	*Lyssa* Length (cm)
		Apex	Body	Root	Apex	Body	Root		
*N. nebulosa*	12	1.8	2.5	3	0.3	0.7	1.2	4 (2 on the right and 2 on the left)	1.7
*P. leo bleyenberghi*	44 ♂	6	7.1	9	1.3	2.5	3.6	7 (3 on the right and 4 on the left)	2.9
	33 ♀	5	5.9	6	0.9	3.0	4.1	5 (3 on the right and 2 on the left)	1.9
*L. lynx*	10	2.6	3.8	4	0.3	0.6	2	9 (5 on the right and 4 on the left)	1.6
*O. manul*	7	1.2	2.2	2	0.2	0.4	0.7	6 (2 on the right and 4 on the left)	1.4

♂—male; ♀—female.

**Table 3:** Measurement (height and width (µm)) of the mechanical filiform papillae and gustatory papillae in the four felid species described in this study.

Species		Mechanical Filiform Papillae					Gustatory Papillae		
		Fi_I_	Fi_II_	Fi_III_	Fi_IV_	Fi_V_	Fu	Vp	Fo
*N. nebulosa*	height	138.75 ± 15.73	1248.16 ± 77.09	541.91 ± 55.08	371.83 ± 157.86	413.75 ± 139.11	647.92 ± 184.55	513.66 ± 51.02	+/−
	width	121.75 ± 20.43	182.91 ± 39.47	254.33 ± 27.64	200.5 ± 41.70	261.5 ± 103.06	407.91 ± 157.26	546.08 ± 93.50	+/−
*P. leo bleyenberghi* (♀ and ♂)	height	181.41 ± 47.25	3650.41 ± 687.53	1567.66 ± 257.97	1041.41 ± 172.64	270.33 ± 48.33	605.91 ± 113.8	2428.58 ± 579.50	−
	width	180.08 ± 25.07	1352.08 ± 90.22	729.33 ± 72.39	278.25 ± 64.68	331.25 ± 70.49	579.08 ± 63.19	2040.25 ± 545.78	−
*L. lynx*	height	88.41 ± 32.57	1106.08 ± 144.06	530.75 ± 160.28	219.16 ± 86.82	367.66 ± 180.89	370.5 ± 79.32	428.28 ± 40.80	−
	width	75.91 ± 22.02	238.25 ± 50.03	232.16 ± 47.12	211.58 ± 61.74	321.91 ± 135.16	464.75 ± 120.30	672.66 ± 121.81	−
*O. manul*	height	124.91 ± 51.25	1651.08 ± 397.87	512.16 ± 74.31	216.75 ± 49.57	257.41 ± 99.56	246.08 ± 73.51	509.5 ± 52.55	457.25 ± 31.13
	width	105.75 ± 18.84	290.91 ± 58.51	196.41 ± 50.67	232.51 ± 43.01	273.08 ± 82.99	284.5 ± 42.12	526.33 ± 227.90	574.91 ± 48.75

Fi_I_, Fi_II_, Fi_III_, Fi_IV_ and Fi_V_—types of filiform papillae; Fu—fungiform papillae; Fo—foliate papillae; Vp—vallate papillae.

## Data Availability

Not applicable.
